# The Association Between Myocardial Strain and Frailty in the Cardiovascular Health Study

**DOI:** 10.1093/geroni/igaa057.1692

**Published:** 2020-12-16

**Authors:** Annabel Tan, Sanjiv J Shah, Jason Sanders, Bruce Psaty, Anne Newman, Chenkai Wu, Julius Gardin, Michelle Odden

**Affiliations:** 1 Stanford University, Stanford, California, United States; 2 Northwestern University, Chicago, Illinois, United States; 3 Brigham And Women’s Hospital, Wellesley, Massachusetts, United States; 4 University of Washington, Seattle, Washington, United States; 5 University of Pittsburgh, Pittsburgh, Pennsylvania, United States; 6 Duke Kunshan University, Kunshan, Jiangsu, China; 7 Rutgers New Jersey Medical School, Hackensack, New Jersey, United States; 8 Stanford University, STANFORD, California, United States

## Abstract

Myocardial strain, measured by speckle tracking echocardiography (STE), is a novel measure of subclinical cardiovascular disease and may reflect myocardial aging. We aimed to explore the association between myocardial strain and frailty, a clinical syndrome of impaired resilience and lack of physiologic reserve. Frailty was defined in 4,042 participants of the Cardiovascular Health Study (CHS) as having 3 or more of the following clinical criteria: weakness, slowness, shrinking, exhaustion, and inactivity. We examined the cross-sectional and longitudinal associations of left ventricular (LV) longitudinal strain, LV early diastolic strain rate and left atrial reservoir strain with frailty in participants with no history of cardiovascular disease or heart failure at the time of echocardiography. In cross-sectional analyses, LV longitudinal strain, LV early diastolic strain, left atrial reservoir strain and LV ejection fraction (measured by conventional echocardiography) levels were lower (worse) among frail participants than among those who were not frail and pre-frail (p<0.01). This association of LV longitudinal strain and frailty was robust to adjustment by LV ejection fraction (adjusted OR: 1.34, 95% CI: 1.20, 2.09). Conversely, LV ejection fraction was not associated with frailty after adjustment for LV longitudinal strain. In longitudinal analyses, LV longitudinal strain and LV early diastolic strain were associated with incident frailty (adjusted OR: 1.49, 95% CI: 1.07, 2.08) and 1.65, 95% CI: 1.15, 2.25, respectively). In community-dwelling older adults without prevalent cardiovascular disease, worse LV longitudinal strain, reflective of subclinical myocardial dysfunction, was associated with frailty independent of LV ejection fraction and other risk factors.

